# The Status Quo of Pharmacogenomics of Tyrosine Kinase Inhibitors in Precision Oncology: A Bibliometric Analysis of the Literature

**DOI:** 10.3390/pharmaceutics16020167

**Published:** 2024-01-25

**Authors:** Abdallah Alzoubi, Hassan Shirazi, Ahmad Alrawashdeh, Arwa M. AL-Dekah, Nadia Ibraheem, Khalid A. Kheirallah

**Affiliations:** 1 Department of Pathological Sciences, College of Medicine, Ajman University, Ajman P.O. Box 346, United Arab Emirates; hassan.shirazi@outlook.com; 2 Department of Pharmacology, Faculty of Medicine, Jordan University of Science and Technology, Irbid 22110, Jordan; 3 Department of Allied Medical Sciences, Faculty of Applied Medical Sciences, Jordan University of Science and Technology, Irbid 22110, Jordan; aaalrawashdeh@just.edu.jo; 4 Kernel Research and Data Analytics, Irbid 22110, Jordan; amaldekah15@sci.just.edu.jo; 5 Department of Public Health and Community Medicine, Faculty of Medicine, Jordan University of Science and Technology, Irbid 22110, Jordan; nadiaibraheem@live.com (N.I.); kkheiral@gmail.com (K.A.K.)

**Keywords:** pharmacogenomics, pharmacogenetics, PGx, tyrosine kinase inhibitors, TKI, bibliometric analysis, precision oncology, personalized medicine

## Abstract

Precision oncology and pharmacogenomics (PGx) intersect in their overarching goal to institute the right treatment for the right patient. However, the translation of these innovations into clinical practice is still lagging behind. Therefore, this study aimed to analyze the current state of research and to predict the future directions of applied PGx in the field of precision oncology as represented by the targeted therapy class of tyrosine kinase inhibitors (TKIs). Advanced bibliometric and scientometric analyses of the literature were performed. The Scopus database was used for the search, and articles published between 2001 and 2023 were extracted. Information about productivity, citations, cluster analysis, keyword co-occurrence, trend topics, and thematic evolution were generated. A total of 448 research articles were included in this analysis. A burst of scholarly activity in the field was noted by the year 2005, peaking in 2017, followed by a remarkable decline to date. Research in the field was hallmarked by consistent and impactful international collaboration, with the US leading in terms of most prolific country, institutions, and total link strength. Thematic evolution in the field points in the direction of more specialized studies on applied pharmacokinetics of available and novel TKIs, particularly for the treatment of lung and breast cancers. Our results delineate a significant advancement in the field of PGx in precision oncology. Notwithstanding the practical challenges to these applications at the point of care, further research, standardization, infrastructure development, and informed policymaking are urgently needed to ensure widespread adoption of PGx.

## 1. Introduction

The Pan American Health Organization and the World Health Organization (PAHO/WHO) have estimated 20 million new cases and 10 million deaths from cancer in 2023 and have projected a rise to 30 million cases by 2040 [1]. Globally, cancer is one of the top two causes of mortality, only behind cardiovascular diseases [2]. With such devastating numbers, research in cancer diagnostics and therapeutics is of the utmost importance. Precision oncology, aided by multi-omics data, including genomics, transcriptomics, and metabolomics, aims to bring the right cancer treatment to the right patient [3]. One cornerstone target chiefly explored in modern precision oncology is related to protein tyrosine kinases, due to their ubiquitous expression and critical role in cell growth, differentiation, adaptation, and response to injury [4]. Currently, at least 90 distinctive protein tyrosine kinase genes have been recognized, 58 of which are encoding for receptor tyrosine kinases (RTKs) [5]. 

Ever since the US Food and Drug Administration (FDA) approval of imatinib for Philadelphia-chromosome-positive chronic myelogenous leukemia in 2001, the growing family of tyrosine kinase inhibitors (TKI) has constantly been perceived as a paradigm-shifting approach to cancer therapy [6]. These agents represented the first step in the direction of cruising away from the conventional nonspecific cytotoxic agents towards targeted therapies of identified molecular abnormalities in cancer cells, i.e., precision oncology [7]. In fact, the advent of TKIs exploited the remarkable understanding of the role of dysregulated RTKs in cancer cell proliferation, and tumor angiogenesis, invasion, and metastasis [8]. There are currently over 50 FDA-approved TKIs in oncology practice for the treatment of hematological and non-hematological cancers [9]. Intriguingly, the majority of these agents have drug-label annotations for “pharmacogenomic biomarkers” from the FDA. 

The rapidly evolving field of pharmacogenomics (PGx) aims at its core to identify a host of actionable genetic variations that influence an individual’s response to medications [10]. By appraising how genetic variations affect drug metabolism, transport, and targets, the overarching goal of PGx is to empower modern personalized medicine approaches, which will enhance the therapeutic efficacy and safety of medications in use [11]. While the term “*pharmacogenomics*” was first coined by Friedrich Vogel in 1959 to insinuate that differences in drug responses can be attributed to genetic variations, it was not until 2005 that the US FDA approved the “AmpliChip CYP450” test to genotype for CYP2D6 and CYP2C19 drug-metabolizing enzymes [12]. Such a landmark has only been possible following the completion of the Human Genome Project in 2003. 

Over the subsequent 20 years, the PGx domain has grown enormously, with a huge body of literature alluding to its analytic and clinical validity, as well as its clinical utility in the fields of psychiatry, infectious diseases, anesthesiology, rheumatology, and oncology, in particular [13]. To that effect, the FDA “Table of Pharmacogenomic Biomarkers in Drug Labeling” has approximately 500 drug label annotations, including indications, dosage, boxed warnings, and precautions [14,15]. An even further extensive archiving of relevant information on the clinical application of PGx has been made public on several other open-source databases, such as the Pharmacogenomics Knowledge Implementation (PharmGKB) and the Clinical Pharmacogenetics Implementation Consortium (CPIC). To date, PharmGKB has 201 clinical guideline annotations and 1018 drug label annotations on 774 drugs annotated [16,17]. 

Despite such a monumental progress and the immensely promising clinical utility of PGx, as well as the significant technological advancements in testing tools, the uptake of PGx into mainstream clinical practice can be judged as “slow and hesitant” at best. Several factors have been implicated in justifying this practical resistance [18]. These include knowledge lagging behind a positive attitude for practice by healthcare professionals, high cost, lack of standardized guidelines, and the yet-to-be addressed ethical and social implications [11,19,20]. For instance, CPIC explicitly states that its published guidelines are only designed to help practitioners “understand how available genetic test results should be used to optimize drug therapy, rather than whether the tests should be ordered.” [21].

Based on this background, this study aims to provide a comprehensive overview of the current state of research on applied PGx in the field of precision oncology as represented by its flagship targeted therapy class of TKIs. The overall objectives were to (1) analyze the productivity, impact, and patterns of the contemporary scholarly activity in the field, and (2) identify the themes, trends, and study hotspots in the field to infer future directions and implications. An advanced bibliometric analysis was used to dissect the available body of literature and to predict the current and future hotspots in research. This innovative tool is well-documented to probe complex relationships among authors, journals, and research fields, thereby facilitating a deeper understanding of academic knowledge, resulting in an improved quantitative capacity in systematic reviews and clinical guideline development compared to the more subjective intuitive assessment methods, such as the peer review [22].

## 2. Materials and Methods

### 2.1. Search Strategy and Data Collection

We searched the Scopus database for relevant articles published between 1 January 2001 and 8 June 2023. Scopus was selected for its comprehensive bibliometric and data mining capabilities, as well as its compatibility with the used software. As the largest abstract and citation database, Scopus combines the key features of PubMed and Web of Science, offering an extensive platform that not only facilitates advanced literature research but also serves academic purposes, including sophisticated citation analysis, making it an optimal choice for in-depth bibliometric studies [23]. Our search strategy included the field codes KEY, Title, and Abstract, and the search query included keywords relevant to (1) pharmacogenomics or pharmacogenetics, (2) cancer, (3) intervention, and (4) tyrosine kinase inhibitors, while excluding studies related to diagnosis and/or screening. We limited the search to articles published in 2001 onwards to focus on contemporary research. Conference papers, conference reviews, editorials, letters, notes, book chapters, short surveys, retracted papers, and non-English papers were excluded by automated document filters. All retrieved studies, along with their associated information, were exported to a CSV file and were then manually screened by two reviewers (A. Alzoubi and H. Shirazi) independently and assessed for eligibility. Any disagreement between the reviewers was resolved through agreement.

The Boolean search strategy used in this study was: “(KEY (pharmacogenetics OR pharmacogenomics) AND KEY (cancer* OR tumor* OR tumour OR neoplas* OR malignan* OR carcinoma OR leukemia* OR leukaemia OR metastat* OR sarcoma* OR teratoma* OR melanoma* OR myeloma*) AND KEY (chemotherapy OR therap* OR *therap OR treatment OR intervention OR drug) AND NOT TITLE (diagnos* OR screen*)) AND TITLE-ABS-KEY (“tyrosine kinase inhibitor” OR tki OR avapritinib OR capmatinib OR pemigatinib OR ripretinib OR selpercatinib OR tucatinib OR entrectinib OR erdafitinib OR fedratinib OR pexidartinib OR upadacitinib OR zanubrutinib OR binimetinib OR dacomitinib OR gilteritinib OR larotrectinib OR lorlatinib OR acalabrutinib OR brigatinib OR neratinib OR alectinib OR cobimetinib OR osimertinib OR ceritinib OR afatinib OR ibrutinib OR trametinib OR bosutinib OR cabozantinib OR ponatinib OR regorafenib OR crizotinib OR vandetanib OR lapatinib OR nilotinib OR dasatinib OR sunitinib OR sorafenib OR erlotinib OR imatinib) AND PUBYEAR > 1999 AND PUBYEAR < 2024 AND (LIMIT-TO (DOCTYPE, “ar”))”.

### 2.2. Scientific Literature Bibliometric Indicators

We exported the final list of included studies to Microsoft Excel and Biblioshiny and calculated the following advanced bibliometric indicators: total number of publications (TP), total citations (TC), average citations (AC), sole-authored publications (SA), co-authored publications (CA), number of contributing authors (NCA), annual collaboration index (ACI), number of cited publications (NCP), citations per cited publication (CCP), collaboration index (CI), collaboration coefficient (CC), number of active years of publication (NAY), productivity per active year of publication (PAY), average citation per year (AC/Y), *h*-index, and *g*-index. These indicators were measured and compared by the year of publication and the accessibility of the journal (traditional [closed] versus open-access). We also identified the ten most prolific contributing countries, institutions, journals, authors, and articles and calculated their relevant indicators.

### 2.3. Visualization Techniques

The Biblioshiny and Visualization of Similarities Viewer (VOSviewer) software version v.1.6.18 were used to visualize the relationship between authors, countries, and author keywords [24]. This relationship is depicted through science mapping. Biblioshiny is a web application enabling access to the Bibliometrix R package non-coders. Bibliometrix provides several tools allowing researchers to carry out comprehensive bibliometric analyses. We conducted a co-authorship of countries’ network visualization, employing VOSviewer to identify the international collaboration countries. We excluded articles authored by more than 25 countries and retained only the countries with a minimum of one published article. No restrictions were placed on the number of citations during the analysis of countries’ co-authorship. In addition, we conducted a co-authorship authors’ network visualization, in which we excluded articles authored by more than 25 authors and retained only the authors with a minimum of two published articles. There was no restriction placed on the number of citations during the analysis of authors co-authorship. Biblioshiny was utilized to visualize author productivity through Lotka’s Law, highlighting the ten most productive authors. 

Further, to detect trending topics related to TKIs, a co-occurrence analysis of the author keywords was performed, considering only those keywords that appeared at least five times in articles while excluding keywords related to countries or regions. A normalization procedure based on the strength of association was applied to eliminate redundancy in similar keywords representing the same concept.

A conceptual structure map encompassing the thematic map and thematic evolution was additionally generated. A plot was created on a two-dimensional matrix using the conceptual network. In this plot, the significance of each theme within the research area as a whole is based on its centrality, as demonstrated by the relevance of the keyword. Additionally, the development of each theme is indicated by its density, as measured by the degree of development [25,26]. Accordingly, themes were distributed into four demarcated quadrants, termed niche, basic, motor, and emerging/declining themes. The conditions for constructing the thematic map in this field included the author’s chosen keywords and a predetermined word count, which was set at 1110. Each individual bubble depicted in the plot represents a distinct network cluster, with the label assigned to the bubble corresponding to the word inside that cluster with the highest frequency value. The relative size of each bubble corresponds to the frequency of the cluster words.

## 3. Results

### 3.1. Bibliometric Analysis of All Articles

The search strategy retrieved 674 articles, of which 226 were excluded by the manual screening. A total of 448 articles were eventually included in the analysis, contributing to a total citation of 21,156 and an average citation of 47.2 per article (Table 1). Then, 183 articles (40.84%) were from closed-access journals while 265 (59.16%) were from open-access journals. Open-access journals demonstrated superior performance across several metrics compared to closed-access journals (Table 1). Compared to closed-access journals, open-access journals exhibited higher TP (264 vs. 183), productivity per active year (15.3 vs. 22.1), TC (6062 vs. 15,094), average citations (33.1 vs. 57.0), and *h*-index (56 vs. 40), but lower *g*-index (116 vs. 71). Open-access journals also showed greater collaboration compared to closed-access journals, with a higher annual collaboration index (9.58 vs. 6.79), collaboration index (10.58 vs. 7.79), and collaboration coefficient (0.91 vs. 0.87).

### 3.2. Annual Publications and Citations Trend

**Figure 1:**
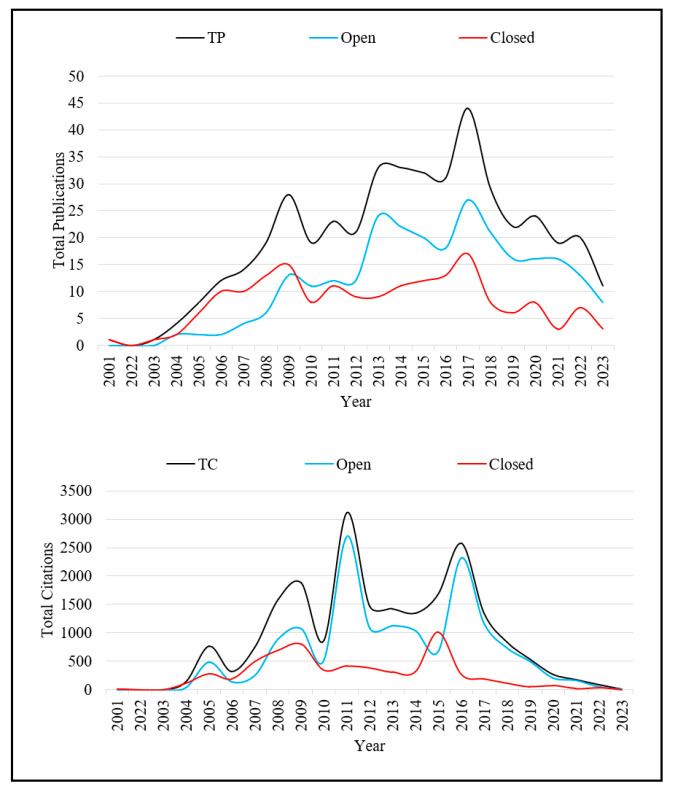
The total number of publications and citations by the year of publication across the type of journals. Abbreviations: TP: Total Publications; TC: Total Citations.

Figure 1 shows the total number of publications and citations over time across the type of journal. Scholarly activity has grown gradually since 2004. There were enormous increases in TP in 2009 and 2013, followed by a noticeable peak in 2017 and a significant decline afterwards. Open-access publications appear to significantly contribute to the surge in TP, particularly after 2010, culminating in the peak in 2017. This implies that the rise in TP can be largely attributed to the growth of open-access journals during this period. Closed-access publications, while also contributing to the TP, show a more tempered increase without the pronounced spikes observed in open-access trends.

During the last two decades, TC experienced significant fluctuations, peaking notably in studies published in 2009, 2011, and 2016. This peak does not align with the peak in TP observed in 2017. Studies published in open-access journals contributed markedly to the citation trend, suggesting their broader reach and impact on the academic community, while studies published in closed-access journals demonstrated a steadier, though less substantial, citation presence. There was a general decline in TC for studies published after 2016 across both open- and closed-access publications, with the decline being more pronounced for closed-access journals.

Supplementary Table S1 details the bibliometrics of all TKI-related articles by the year of publication. All bibliometrics had improved over the years corresponding to the total number of publications and citations. The citation average ranged between 0.55 in 2023 and 95.50 in 2005. Sole-authored articles were 25 (ranged between 0 for several years and 4 in 2005 and 2010), while co-authored publications were 423 (ranged between 0 in 2003 and 44 in 2017). The number of contributing authors was 4232 and ranged between 1 in 2003 and 556 in 2017. In addition, the annual collaboration index (ACI) was 8.45, and ranged between 0 for articles in 2003 and 12.40 for articles in 2022. The collaboration coefficient was 0.89 and ranged between 0 for articles published in 2003 and 0.93 for articles published in 2022. 

Further, a total of 448 publications were cited, gathering a total of 21,156 citations. The number of cited publications ranged from 1 article in 2001 and 2003 to 44 articles in 2017. Finally, the *h*-index for all TKI-related articles was 70 and the *g*-index was 130, indicating significant impact and consistency of the publications.

### 3.3. The Most Prolific Countries and International Collaborations

**Figure 2:**
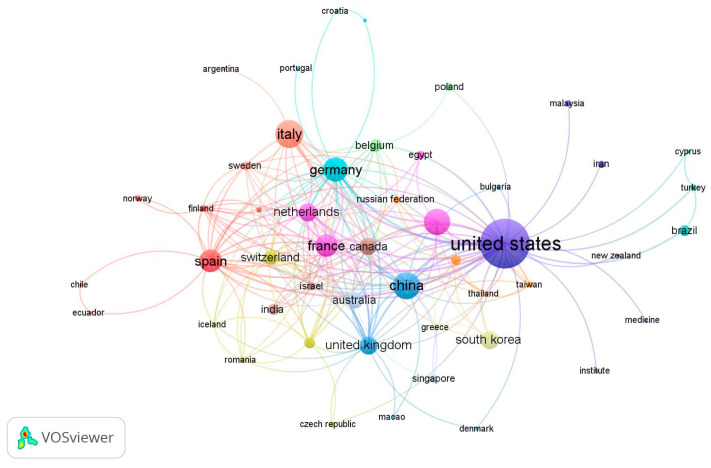
Network visualization map for country co-authorship (international collaboration).

Table 2 displays a list of the top 10 countries out of 59 countries, each with a minimum of five articles. The extent of international collaboration between all countries is illustrated in the VOSviewer network visualization map, represented by the Total Link Strength (TLS) between countries (Figure 2). The United States emerged as the most prolific contributor country, with 166 articles (37.1%), followed by Italy with 53 articles (11.8%), and China with 50 articles (11.2%). Regarding international collaborations, the United States topped the chart with a TLS of 93, followed by China with a TLS of 22, as depicted in Figure 2 and Table 2.

### 3.4. The Most Prolific Institutions, Journals, and Authors

Table 3 lists the top 10 institutions out of 59 countries based on the total number of publications. Ohio State University was the most productive institution, with 61 articles (13.6%), followed by the US National Cancer Institute with 52 articles (11.6%) and the University of Chicago with 43 articles (9.6%). A total of 10 institutions had published at least 31 articles each. Interestingly, six of the top ten institutions are in the United States.

Table 4 presents the top 10 most productive journals out of 224 journals. Clinical Cancer Research has published the most; 21 articles with 2826 citations. The journal’s average citation is 134.57. The next journal, *Cancer Chemotherapy and Pharmacology*, has 15 articles and 714 citations, averaging 47.60 per article. The journals *Oncotarget* and *Lung Cancer* have 13 and 12 articles, generating 357 and 349 citations, respectively. Further, the collaboration coefficient for most journals ranged from 0.87 to 0.92, indicating a high degree of collaborative effort. For instance, the journal *Pharmacogenomics* has a total of nine co-authored publications with 77 contributing authors. 

**Figure 3:**
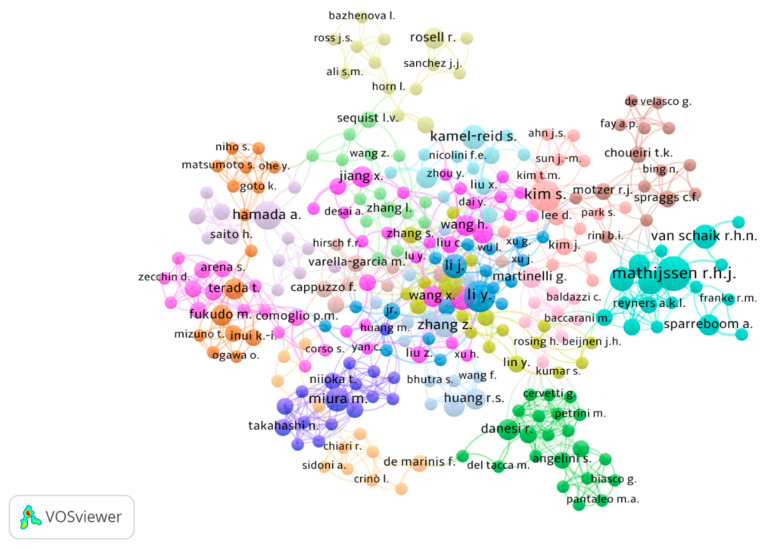
VOSviewer network of author co-authorship map weighted by the number of publications. Note: because some names may overlap, others may not be shown.

To explore the relationship between authors and the number of published articles, the distribution of author productivity and the number of articles, based on Lotka’s law, was computed using Biblioshiny. The majority of authors (n = 3161) had one article, 438 authors had two to five articles, while 7 authors had fewer than ten articles. Table 5 shows the top 10 authors out of 3606 authors, ranked based on the total number of publications (TP). Each of the top 10 most published authors has contributed five or more articles on the topic (range: five to nine). Li, Y. emerged as the most productive author (TP = 9; 2.0%), followed by Mathijssen, R.H.J. (TP = 8; 1.8%), and Zhang, Z. (TP = 8; 1.8%). Regarding the total citations (TC) and author impact indices, taking the career length into consideration, Zhang, Z. emerged as the most impactful author (TC = 381; *h*-index = 5; *m*-index = 0.714), followed by Mathijssen, R.H.J. (TC = 374; *h*-index = 7; *m*-index = 0.389) and Hamada, A. (TC = 279; *h*-index = 5; *m*-index = 0.385). To enhance the bibliometric results for the most productive author, we conducted network visualization mapping (Figure 3).

### 3.5. The Most Cited Articles

Table 6 shows the top 10 most influential articles, based on the total number of citations (TC). These articles represented nearly 30.95% of the total citations (6548 out of 21,156). The top-cited article was entitled “Subtypes of pancreatic ductal adenocarcinoma and their differing responses to therapy”, with a TC of 1131 citations. The study “A Landscape of Pharmacogenomic Interactions in Cancer” and the study “EGFR mutations and ALK rearrangements are associated with low response rates to PD-1 pathway blockade in non-small cell lung cancer: A retrospective analysis” came in second (TC = 1043) and third place (TC = 884), respectively.

### 3.6. Keywords Analysis, Trend Topics, and Thematic Evolution

**Figure 4:**
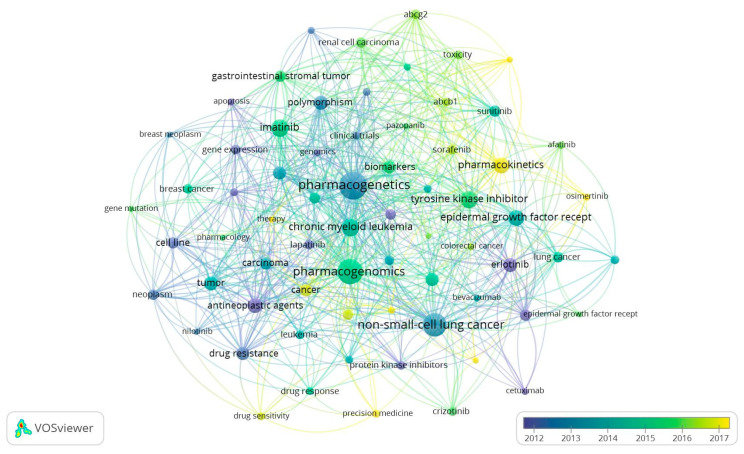
The most frequent keywords (Trend Topics), as determined by a co-occurrence analysis of author keywords.

Figure 4 illustrates the trending topics related to TKIs. Among the 1108 keywords analyzed, 68 met the defined threshold. Keywords were grouped into six distinct clusters if they had a minimum occurrence of ≥5 times. The most frequently occurring keywords were “pharmacogenetics” (co-occurrences = 101), “pharmacogenomics” (co-occurrences = 80), “non-small-cell lung cancer” (co-occurrences = 71), “tyrosine kinase inhibitors” (co-occurrences = 41), “imatinib” (co-occurrences = 40), and “chronic myeloid leukemia” (co-occurrences = 39). According to the color scale in Figure 4, which shows the year of publication, most of these trending topics appeared in publications from 2012 to 2016.

**Figure 5:**
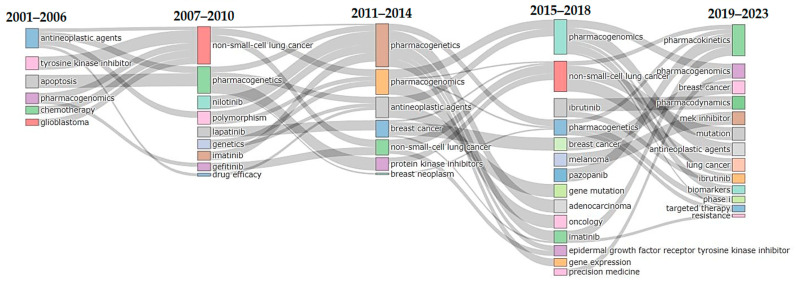
Thematic evolution of each topic and theme under five time slices. A longitudinal representation is adopted to facilitate understanding of the tendencies of certain topics to merge with other themes or split into several other themes over the study period.

To further elucidate the thematic evolution within the research field under study, alluvial graphs were generated by dividing the time span into different time slices. Based on the distribution of articles per year, we identified five time slices, with four cutting points set at the years 2006, 2010, 2014, and 2018. As shown in Figure 5, in the first time slice of 2001–2006, most extracted articles focused on the overall themes of antineoplastic agents, TKIs, and apoptosis. In the second time slice (2007–2010), studies emphasized the application of PGx in the treatment of NSCLC. In the third time slice (2011–2014), breast cancer emerged as a new theme in relation to PGx and antineoplastic agents. In the fourth time slice (2015–2018), research continued to emphasize PGx in NSCLC, with an increased focus on the drug ibrutinib. In the last time slice (2019–2023), studies had a more oriented exploration of TKIs pharmacokinetics and pharmacodynamics, PGx, and breast cancer.

### 3.7. Thematic Map of the Field

**Figure 6:**
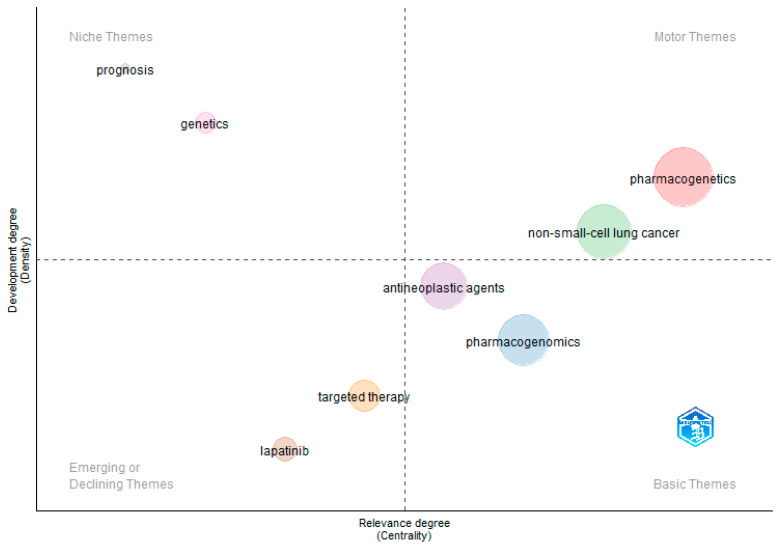
Thematic map of the field.

The primary goal of this examination was to intuitively analyze the development of themes in TKI-related research between 2001 and 2023. A plot was generated on a two-dimensional matrix using the conceptual network, and divided into four distinct quadrants based on the centrality and density of themes (Figure 6). The quadrant in the upper-left region, labeled as ‘*Niche Themes*’, pertains to themes that were extensively developed but remained isolated. These themes had strong internal connections but lacked external connectedness, resulting in relatively limited significance. Themes elucidated in this quadrant were related to genetics and prognostics (Figure 6). The quadrant in the lower-left region, referred to as ‘*Emerging or Declining Themes*’, exhibits themes with low density and centrality indicating a relatively poor level of development. Themes found in this quadrant included targeted therapy and the drug lapatinib (Figure 6). The quadrant in the upper-right section, referred to as ‘*Motor Themes*’, encompasses themes that exhibited significant density and centrality, interconnected with other closely related themes, indicating substantial development and major relevance within the realm of research. Themes elucidated in this quadrant were pharmacogenetics and NSCLC (Figure 6). Finally, the lower-right quadrant, referred to as ‘*Basic Themes*’, encompasses primary and transversal themes that are of utmost importance for study but have not been fully developed yet [27]. Themes displayed in this quadrant were pharmacogenomics and antineoplastic agents (Figure 6).

## 4. Discussion

This bibliometric analysis has shed light on the current state of research on applied PGx in the field of precision oncology, with TKIs being deployed as the representative class of targeted cancer therapies. The major findings of this study are: (1) a burst of scholarly activity in the field was noted by the year 2005, peaking in 2017, followed by a remarkable decline to date; (2) research in the field is hallmarked by significant international collaboration, impact, and consistency, with the US leading the charts in terms of most prolific country, institutions, and TLS; and (3) thematic evolution in the field points in the direction of more specialized studies on the applied pharmacokinetics and pharmacodynamics of available and novel TKIs, particularly for the treatment of NSCLC and breast cancers. 

The conclusion of the Human Genome Project in 2003 allowed for significant advancements and sustainable growth in the fields of PGx and precision oncology [28]. The two fields intricately overlap in their evolution trajectories, since oncology represents the domain where PGx finds its most valuable contribution [29]. By the year 2005, the enormous technological innovations in genomic testing tools enabled researchers to explore a wide array of molecular targets in oncology, including RTKs [30]. This explains the noted surge in research productivity and the rising wave of novel targeted therapies introduced at the time. Indeed, the pioneering and expansion of TKIs in oncology practice during this period may be regarded as a chief illustration of a revolutionary transformation in cancer therapeutics from the nonspecific cytotoxic agents onto therapies targeting specific identified molecular abnormalities. 

Receptor and non-receptor protein tyrosine kinases have been found to account for more than half of the human protooncogene and oncogene products, which can drive carcinogenesis once dysregulated [31]. Imatinib (*Gleevec*) was the first marketed TKI for the treatment of chronic myelogenous leukemia in 2001, and there are currently more than 50 other TKIs in practice, most of which are targeted cancer therapies [6]. These TKIs can be divided into epithelial growth factor receptor (EGFR) inhibitors, vascular endothelial growth factor receptor (VEGFR) inhibitors, anaplastic lymphoma kinase (ALK) inhibitors, and BCR-ABL inhibitors [5]. Substantial evidence signifies TKI superiority over conventional cytotoxic drugs in terms of selectivity, efficacy, and safety in the treatment of hematological (such as leukemias and lymphomas) and non-hematological cancers (such as NSCLC, renal cell carcinoma, and breast cancers) [32,33,34]. 

Intriguingly, targeted cancer therapies, such as TKIs, are mostly annotated for PGx labelling by the FDA and CPIC, notwithstanding the unremitting challenges in the application of PGx in mainstream clinical practice. These PGx annotations are principally related to patient selection, dosing, administration, precautions, and adverse effects. Such extensive labelling is directly linked to the observed limitations and shortcomings of TKI therapy in cancer. For instance, previous studies have shown that the median effective time for TKI therapy is limited to 5–9 months, due to the emergence of acquired resistance [35]. Almost 50% of patients with NSCLC with documented resistance to TKIs were positive for T790M mutations [36], while 20% were positive for c-MET gene amplification [37]. With PGx testing in place, physicians have an invaluable window into selecting the most effective and least resistant therapy for the right patient. 

Our bibliometric analysis additionally reveals a steep downward trend in original experimental research productivity after 2017. A few explanations exist for this depreciation in total publications and citations, such as the surfacing of the aforementioned drug resistance [9], the emergence of monoclonal antibodies for targeted cancer therapy [38], or unforeseen issues related to the safety profile of TKIs [39,40]. Perhaps the declining productivity could also be attributed to the saturation of research in the field, the inadequate applicability of PGx in clinical practice, or more simply the cyclical waxing and waning nature of research interests. It must be noted, however, that despite the net positive trend seen in Figure 2, the peak of 44 articles published in 2017 is still representative of a large discrepancy, particularly when compared with other such studies [41,42].

Further, we have gauged the conceptual structure of the field through analyses of the research trends and hotspots, as well as the co-occurrences of author keywords in the retrieved literature. Metrics like the centrality and density of keywords were utilized to outline the research themes, and resulted in the identification of eight distinct thematic clusters that were micro-examined within the four quadrants of a thematic map (Figure 6), as follows:

- Clusters 7 and 8 (prognostics and genomics) were identified as niche themes, indicating their low significance in the field, having less relevant ties with other themes. The most recent studies within those clusters discussed drug responses and genomic data from a multi-omic perspective [43,44].
- Clusters 1 and 3 (pharmacogenetics and NSCLC) were identified as motor themes, due to their high occurrence in research studies, and were thus perceived to be making rapid advancements in the TKI domain. The most recent studies in those clusters investigated the impact of concurrent drug–drug-interactions on imatinib response in patients with gastrointestinal stromal tumors [45] and the mechanisms of drug resistance in patients with advanced or refractory lung cancer [46].
- Clusters 5 and 6 (targeted therapy and lapatinib) were identified as emerging/declining themes, suggesting a somewhat limited and marginalized position. The most recent studies in those clusters explored novel tools to predicts drug responses in pancreatic ductal adenocarcinoma [47] and colorectal cancer [48].
- Clusters 2 and 4 (PGx and antineoplastic agents) were identified as basic themes, due to their potential importance to the field; however, they were perceived to need more tangible development. The most recent studies in those clusters lay the foundation for future integrative analyses of PGx data in different tumor contexts for the generation of a ‘*pancancer*’ treatment [49], and the utilization of PGx in the identification of new antineoplastic treatments for head and neck squamous cell carcinoma [50].

Although bibliometric analyses provide a multidimensional view of the global research trends over time, they in general are subject to certain limitations. With the core of bibliometrics residing in citation counts, it is important to note that the citation counts of a body of literature indicate its impact and international recognition rather than its true quality, and could vary based on discipline, authors, and type of publication. In addition, owing to a lower citation count, works in the literature published more recently can be inadvertently omitted from the top ten lists. This is further exacerbated by the use of Scopus as the sole search engine. Due to the lag times in updating results, the most current or “*in-press*” publications may also be excluded. Furthermore, machine algorithms used to narrow search results may generate unknown and unforeseen biases, although this was overcome by the manual scanning of the whole retrieved literature. A limitation specific to the present study involves the exclusion of all non-English publications, as well as review articles and case reports, which can possibly dismiss some high-impact literature. Although VOSviewer is an indispensable tool in a statistician’s arsenal, it inherently lacks the ability to analyze full text papers; instead, it relies on various author metrics which may not be entirely representative of the author’s vision. Moreover, conducting a weighted analysis of the literature based on quality assessment was beyond the scope of this study, and therefore equal attention may have been given to publications of differing quality. Lastly, it is perhaps imperative to recognize the indisputable shortcoming in this kind of research that minor errors in author names or institutional affiliations or journal data (volume, series, issue, pages) could complicate analyses and affect the study outcomes. In spite of the aforementioned limitations, bibliometric analyses are an irreplaceable instrument to gain a snapshot of the most current research hotspots in a given field. 

## 5. Conclusions

In conclusion, this study appraised the status quo of research on applied PGx of TKIs in precision oncology through a comprehensive bibliometric analysis. While our results delineate a significant advancement in the field, hallmarked by consistent and impactful international collaborative efforts, translation into real-world clinical practice is still lagging behind and sensible challenges are there to be overcome in the near future. The current opportunities presented with artificial intelligence and machine learning techniques can aid in the interpretation of complex genetic data and facilitate their integration into precision oncology practice. Further research, standardization, infrastructure development, and informed policymaking are urgently needed to ensure the widespread adoption of PGx.

## Figures and Tables

**Table 1:** Summary of the bibliometric analysis results on all publications and by the type of journal: closed- versus open-access journals.

Metric	All Publications	Closed-Access Journals	Open-Access Journals
Total publications	448	183	265
Productivity per active year	37.33	15.25	22.08
Total citations	21,156	6062	15,094
Average citations	47.22	33.13	56.96
Number of cited publications	421	166	255
Citations per cited publication	50.25	36.52	59.19
*h*-index	70	40	56
*g*-index	130	116	71
Sole-authored publications	25	18	7
Co-authored publications	423	165	258
Number of contributing authors	4232	1425	2804
Annual collaboration index	8.45	6.79	9.58
Collaboration index	9.45	7.79	10.58
Collaboration coefficient	0.89	0.87	0.91

**Table 2:** A bibliometric analysis of the top 10 most prolific countries.

Rank	Country	TP	TC	AC	TLS
1	United States	166	13,724	82.67	93
2	Italy	53	1846	34.83	20
3	China	50	2801	56.02	22
4	Japan	46	2224	48.35	12
5	Germany	39	2998	76.87	20
6	France	36	2934	81.50	16
7	Spain	36	3233	89.81	20
8	Netherlands	23	1891	82.22	12
9	United Kingdom	23	2670	116.09	18
10	Republic of Korea	22	1155	52.50	11

Abbreviations: TP: total number of publications; TC: total citations; AC: average citations; TLS: total link strength.

**Table 3:** A bibliometric analysis of the top 10 most prolific institutions.

Rank	Institution	Number of Articles (%TP)	Country
1	Ohio State University	61 (13.6)	United States
2	National Cancer Institute	52 (11.6)	United States
3	University of Chicago	43 (9.6)	United States
4	Dana-Farber Cancer Institute	41 (9.2)	United States
5	Harvard Medical School	37 (8.3)	United States
6	Mayo Clinic	36 (8.0)	United States
7	University of Pisa	33 (7.4)	Italy
8	University of Bologna	33 (7.4)	Italy
9	Sungkyunkwan University School of Medicine	32 (7.1)	Republic of Korea
10	Oslo University Hospital	31 (6.9)	Norway

Abbreviations: TP: total number of publications.

**Table 4:** A bibliometric analysis of the top 10 most prolific journals.

Rank	Journal	TP	TC	AC	SA	CA	NCA	ACI	NCP	CCP	CI	CC	*h*	*g*	NAY	PAY
1	*Clinical Cancer Research*	21	2826	134.57		21	252	11.00	21	134.57	12.00	0.92	19	21	11	1.91
2	*Cancer Chemotherapy and Pharmacology*	15	714	47.60		15	144	8.60	15	47.60	9.60	0.90	12	15	11	1.36
3	*Oncotarget*	13	357	27.46		13	152	10.69	13	27.46	11.69	0.91	11	13	6	2.17
4	*Lung Cancer*	12	349	29.08		12	111	8.25	12	29.08	9.25	0.89	10	12	9	1.33
5	*Pharmacogenomics*	10	224	22.40	1	9	77	6.70	9	24.89	7.70	0.87	6	10	8	1.25
6	*Journal of Thoracic Oncology*	9	460	51.11		9	97	9.78	9	51.11	10.78	0.91	9	9	9	1.00
7	*Pharmacogenomics Journal*	8	263	32.88		8	69	7.63	8	32.88	8.63	0.88	6	8	8	1.00
8	*PLoS ONE*	8	494	61.75		8	95	10.88	8	61.75	11.88	0.92	6	8	5	1.60
9	*European Journal of Cancer*	6	180	30.00		6	67	10.17	6	30.00	11.17	0.91	5	6	5	1.20
10	*Investigational New Drugs*	6	139	23.17		6	74	11.33	6	23.17	12.33	0.92	5	6	4	1.50

Abbreviations: TP: total number of publications; TC: total citations; AC: average citations; SA: sole-authored publications; CA: co-authored publications; NCA: number of contributing authors; ACI: annual collaboration index; NCP: number of cited publications; CCP: citations per cited publication; CI: collaboration index; CC: collaboration coefficient; *h*: *h*-index; *g*: *g*-index; NAY: number of active years of publication; PAY: productivity per active year of publication.

**Table 5:** A bibliometric analysis of the top 10 most prolific authors.

Rank	Authors	TP	TC	AC	*h*-Index	*g*-Index	*m*-Index	PY-Start
1	Li, Y.	9	335	37.22	5	9	0.333	2009
2	Mathijssen, R.H.J.	8	374	46.75	7	8	0.389	2006
3	Zhang, Z.	8	381	47.63	5	8	0.714	2017
4	Kim, S.	7	121	17.29	5	7	0.333	2009
5	Hamada, A.	6	279	46.50	5	6	0.385	2011
6	Miura, M.	6	210	35.00	5	6	0.357	2010
7	Wang, Y.	6	41	6.83	3	6	0.375	2016
8	Gelderblom, H.	5	271	54.20	5	5	0.333	2009
9	Guchelaar, H.J.	5	276	55.20	5	5	0.333	2009
10	Kamel-Reid, S.	5	435	87.00	5	5	0.333	2009

Abbreviations: TP: total number of publications; TC: total citations; AC: average citations; PY: publication year.

**Table 6:** A bibliometric analysis of the top 10 most cited articles.

Rank	First Author	Journal	Year	TC	TC/Y	NCA	Normalized TC
1	Collisson, E.A.	*Nature Medicine*	2011	1131	87.00	18	8.35
2	Iorio, F.	*Cell*	2016	1043	130.38	39	12.54
3	Gainor, J.F.	*Clinical Cancer Research*	2016	884	110.50	21	10.63
4	Yang, J.C-H.	*Lancet Oncology*	2015	707	78.56	13	13.47
5	Carvajal, R.D.	*JAMA*	2011	664	51.08	18	4.90
6	Crystal, A.S.	*Science*	2014	550	55.00	26	13.47
7	Yauch, R.L.	*Clinical Cancer Research*	2005	473	24.89	14	4.95
8	Luo, B.	*PNAS*	2008	431	26.94	24	5.22
9	Heiser, L.M.	*PNAS*	2012	344	28.67	43	4.91
10	Hidalgo, M.	*Molecular Cancer Therapeutics*	2011	321	24.69	10	2.37

Abbreviations: TC: total citations; NCA: number of contributing authors; TC/Y: total citation per year.

## Data Availability

Data presented in this study are included in the article/supplementary material; further inquiries can be directed to the corresponding author.

## Supplementary Materials

The following supporting information can be downloaded at: https://www.mdpi.com/article/10.3390/pharmaceutics16020167/s1, Table S1: Bibliometric parameters for all retrieved TKI-related articles by the year of publication.
