# Supplementary material for: The Status Quo of Pharmacogenomics of Tyrosine Kinase Inhibitors in Precision Oncology: A Bibliometric Analysis of the Literature
Source: Pharmaceutics. 2024 Jan 25;16(2):167. doi: 10.3390/pharmaceutics16020167 (PMC10892459; doi:10.3390/pharmaceutics16020167)
Supplement: Supplementary file 1 [file pharmaceutics-16-00167-s001.zip › pharmaceutics-2853572-supplementary.pdf]

Supplementary Table 1: Bibliometric parameters for all retrieved TKI-related articles by the year of publication

year	TP	TC	AC	SA	CA	NCA	ACI	NCP	CCP	PCP	CI	CC	*h*	*g*	*i10*
**2001**	1	12	12.00	-	1	3	2.00	1	12.00	1.00	3.00	0.67	1	1	0
**2003**	1	1	1.00	1	0	1	-	1	1.00	1.00	1.00	-	1	1	0
**2004**	4	137	34.25	-	4	18	3.50	4	34.25	1.00	4.50	0.78	4	4	3
**2005**	8	764	95.50	4	4	26	2.25	7	109.14	0.88	3.25	0.69	6	8	5
**2006**	12	320	26.67	2	10	44	2.67	11	29.09	0.92	3.67	0.73	9	12	9
**2007**	14	751	53.64	2	12	98	6.00	12	62.58	0.86	7.00	0.86	9	14	9
**2008**	19	1,570	82.63	3	16	139	6.32	17	92.35	0.89	7.32	0.86	14	19	15
**2009**	28	1,879	67.11	2	26	231	7.25	26	72.27	0.93	8.25	0.88	19	28	20
**2010**	19	855	45.00	4	15	122	5.42	17	50.29	0.89	6.42	0.84	15	19	16
**2011**	23	3,115	135.43	2	21	178	6.74	22	141.59	0.96	7.74	0.87	18	23	21
**2012**	21	1,472	70.10	1	20	195	8.29	20	73.60	0.95	9.29	0.89	16	21	19
**2013**	33	1,425	43.18	2	31	269	7.15	33	43.18	1.00	8.15	0.88	21	33	29
**2014**	33	1,347	40.82	-	33	335	9.15	32	42.09	0.97	10.15	0.90	20	33	30
**2015**	32	1,680	52.50	-	32	336	9.50	32	52.50	1.00	10.50	0.90	19	23	32
**2016**	31	2,579	83.19	-	31	297	8.58	30	85.97	0.97	9.58	0.90	18	31	27
**2017**	44	1,348	30.64	-	44	556	11.64	43	31.35	0.98	12.64	0.92	17	36	11
**2018**	29	836	28.83	-	29	357	11.31	29	28.83	1.00	12.31	0.92	15	28	18
**2019**	22	539	24.50	-	22	224	9.18	22	24.50	1.00	10.18	0.90	12	22	14
**2020**	24	266	11.08	1	23	228	8.50	24	11.08	1.00	9.50	0.89	10	15	10
**2021**	19	173	9.11	1	18	179	8.42	19	9.11	1.00	9.42	0.89	6	12	4
**2022**	20	81	4.05	-	20	268	12.40	16	5.06	0.80	13.40	0.93	4	8	3
**2023**	11	6	0.55	-	11	128	10.64	3	2.00	0.27	11.64	0.91	2	2	0
**Total**	**448**	**21,156**	**47.22**	**25**	**423**	**4,232**	**8.45**	**421**	**50.25**	**0.94**	**9.45**	**0.89**	**70**	**130**	**302**

Abbreviations: TP: total number of publications; TC: total citations; AC: average citations; SA: sole-authored publications; CA: co-authored publications; NCA: number of contributing authors; ACI: annual collaboration index; NCP: number of cited publications; CCP: citations per cited publication; PCP: proportion of cited publications; CI: collaboration index; CC: collaboration coefficient; h: h-index; g: g-index; i10: i10-index.
